# Kinase Inhibitors with Antiepileptic Properties Identified with a Novel in Vitro Screening Platform

**DOI:** 10.3390/ijms20102502

**Published:** 2019-05-21

**Authors:** Jing Liu, Madison Schenker, Shabnam Ghiasvand, Yevgeny Berdichevsky

**Affiliations:** 1 Department of Electrical and Computer Engineering, Lehigh University, Bethlehem, PA 18015, USA; Jing.Liu2@ucsf.edu; 2 Department of Bioengineering, Lehigh University, Bethlehem, PA 18015, USA; mdschenker@gmail.com (M.S.); shg515@lehigh.edu (S.G.); 3 Department of Bioengineering and Department of Electrical and Computer Engineering, Lehigh University, Bethlehem, PA 18015, USA

**Keywords:** epilepsy, seizure, epileptogenesis, kinase, receptor tyrosine kinase, organotypic, phenotypic screen, src family kinases, TrkB, cFMS inhibitor

## Abstract

Kinase signaling plays an important role in acquired epilepsy, but only a small percentage of the total kinome has been investigated in this context. A major roadblock that prevents the systematic investigation of the contributions of kinase signaling networks is the slow speed of experiments designed to test the chronic effects of target inhibition in epilepsy models. We developed a novel in vitro screening platform based on microwire recordings from an organotypic hippocampal culture model of acquired epilepsy. This platform enables the direct, parallel determination of the effects of compounds on spontaneous epileptiform activity. The platform also enables repeated recordings from the same culture over two-week long experiments. We screened 45 kinase inhibitors and quantified their effects on seizure duration, the frequency of paroxysmal activity, and electrographic load. We identified several inhibitors with previously unknown antiepileptic properties. We also used kinase inhibition profile cross-referencing to identify kinases that are inhibited by seizure-suppressing compounds, but not by compounds that had no effect on seizures.

## 1. Introduction

Characteristics of the epileptic brain include axon sprouting, synaptic reorganization, inflammation, and hyperexcitability [1,2]. Axon and dendritic growth and development, synaptic formation and maintenance, changes in receptor number and composition, and inflammatory processes are regulated by cell signaling pathway networks [2,3,4,5,6,7,8,9]. The inhibition of these pathways has the potential to prevent the formation of epileptic circuitry and thus prevent epilepsy development after brain injury, or even disrupt existing epileptic circuits and have a permanent disease-modifying effect. In contrast, existing anticonvulsants do not prevent or cure epilepsy.

**Figure 1:**
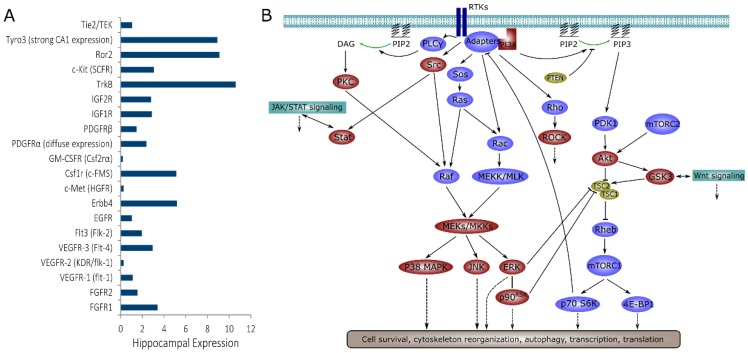
Receptor tyrosine kinase (RTK) signaling in the hippocampus. (**A**) Level of RTK expression in the hippocampus, based on Allen Brain Atlas mouse data (www.brain-map.org). Most RTKs were concentrated in the pyramidal layers of the hippocampus with the exception of PDGFRα, which had strong but diffuse expression; (**B**) Downstream signaling activated by RTKs. Kinases that were inhibited in this work are highlighted in red. Cross-talk with JAK/STAT and Wnt signaling is also shown.

Kinase signaling, including activation of PI3K-Akt-mTOR, JAK-STAT, and BDNF-TrkB pathways, has been implicated in animal and in vitro models of acquired chronic epilepsies [10,11,12,13,14,15,16,17]. However, kinases that have been found to play a role in epilepsy to date represent only a small percentage of the total kinome, since the human genome includes over 500 kinase genes [18]. Many of these kinases play important roles in neurons, glia, and microglia [19], and there is a significant likelihood that they may be involved in epileptogenesis and/or the progression of epilepsy. Small molecule inhibitors have been synthesized for many of these kinases, and used extensively in cancer research, with some inhibitors successfully passing clinical trials [20]. While kinase inhibitors are not always specific, their inhibition profiles have been experimentally determined and described [21,22,23]. Despite the availability of inhibitors, only a few kinases have been investigated with the goal of preventing or modifying epilepsy. For example, receptor tyrosine kinases (RTKs), a class of kinases that act as cell surface receptors, include FGFRs, VEGFRs, Flt, EGFR, Erbb receptors, IGF-1R, c-Met, cFMS, GM-CSFR, and PDGFRs as well as neurotrophin receptors (TrkB and others). All of the RTKs listed above (and their ligands) are expressed in the brain, and changes in their expression or phosphorylation have been reported in the injured or epileptic brain [24,25,26,27,28,29,30,31]. However, only a few non-neurotrophin RTKs, such as IGF-1R, have been explored as potential targets for antiepileptic drugs [32]. The RTK signaling network is highly complex. At least 20 RTKs are expressed in the hippocampus at significant levels [33], and their downstream signaling includes multiple pathways such as PI3K-Akt-mTOR, multiple MAPKs, Stat, and Rho-ROCK (Figure 1) [34,35,36,37,38,39,40,41]. Downstream kinases, with the exception of those involved in mTOR signaling, have also been under-explored as antiepileptic drug targets.

A major roadblock that prevents the systematic investigation of the contributions of RTKs and their signaling network to epilepsy is the slow speed of experiments designed to test the chronic effects of target inhibition in animal models of epilepsy. We have recently reported a novel in vitro antiepileptic drug screening platform that integrated an organotypic hippocampal culture model of epilepsy with microfluidic-multiple electrode array (μflow-MEA) chips [42]. The platform was capable of monitoring chronic epileptiform activity in inhibitor-treated cultures. A pilot screen conducted with the μflow-MEA platform revealed antiepileptic effects of EGFR/ErbB-2 and cFMS inhibitors. However, the transferability of the platform to other laboratories was limited due to complex clean room microfabrication requirements of the μflow-MEA chips. Here, we report a simplified screening platform that is based on standard tissue culture plates and commercially available microwire electrodes. We used this platform to evaluate the effects of 45 inhibitors of RTKs and their signaling pathways (Table 1) on the epileptiform activity in organotypic hippocampal cultures. We then used the data on kinase inhibitor selectivity to determine the likely kinase targets of the efficacious inhibitors.

## 2. Results

**Figure 2:**
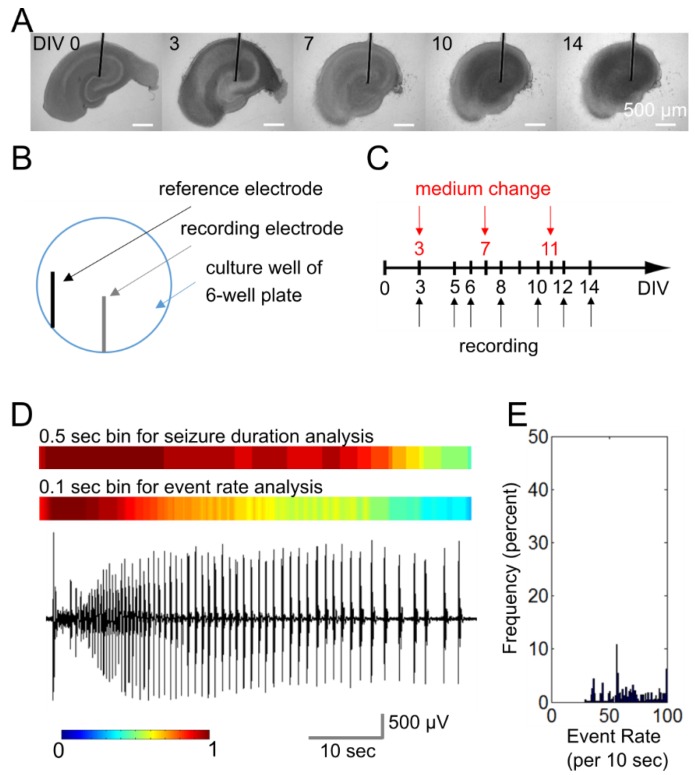
Microwire-based platform for antiepileptic drug screening. (**A**) Organotypic hippocampal culture maintained on a microwire for two weeks. Scale bars, 500 µm; (**B**) The electrode layout in a culture well of a 6-well plate; (**C**) Recording and medium change schedule for a two-week experiment; (**D**) Representative recordings of an ictal-like (electrographic seizure) event. Color plots above the recording show 10-s sliding window results using 0.5-sec and 0.1-sec bins (a value of 1 means that all of the bins in the 10-sec window were above the threshold); (**E**) Histogram shows the distribution of event rates (in a 10-sec sliding window) in this recording.

**Figure 3:**
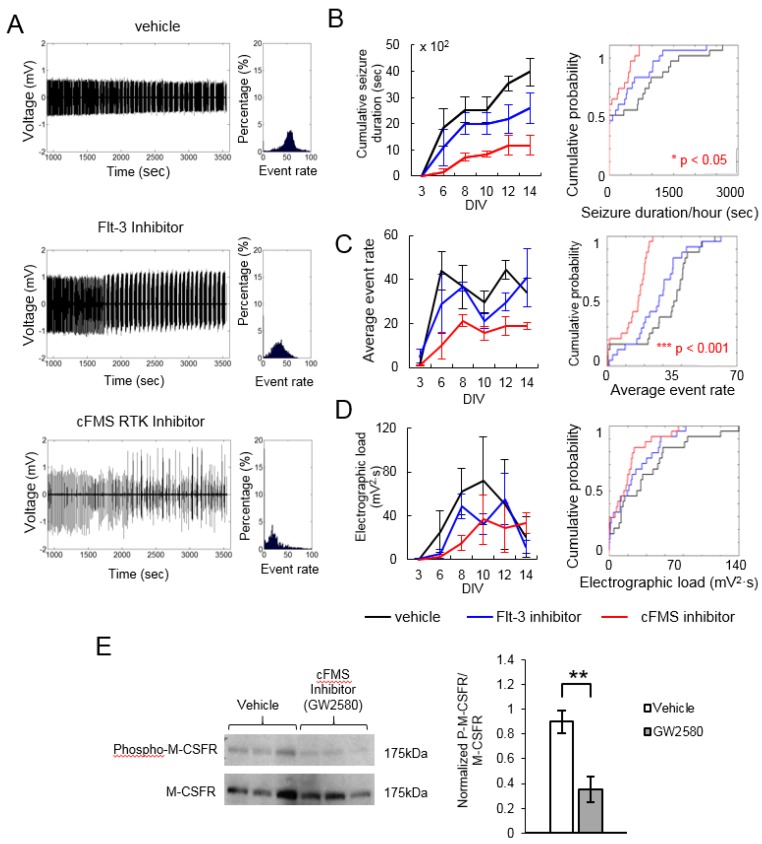
Examples of data obtained with microwire-based platform. (**A**) Representative recordings at 12 days in vitro (DIV). Histogram on the right shows the distribution of event rates (in 10-s bins) in a one-hour recording; (**B**) Plot on the left shows cumulative seizure duration in *n* = 4 cultures treated with either vehicle cFMS Inhibitor (GW2580, 5 µM) or Flt-3 Inhibitor (2 µM) versus DIV. Plot on the right shows the cumulative probability of seizure durations per one hour of recording in combined recording data; (**C**) Average event rate versus DIV (left) and as cumulative probability (right); (**D**) Average electrographic load versus DIV (left) and as cumulative probability (right). *p* is from Kolmogorov–Smirnov test on combined data per condition, error bars indicate standard deviation; (**E**) Western blot analysis validating the inhibition of cFMS kinase. Left, the upper bar shows the inhibition of M-CSFR (cFMS) phosphorylation in cultures treated with cFMS inhibitor GW2580. The bottom bar represents the presence of m-CSF in hippocampal cultures. Right, the expression level of phospho-M-CSF between control and cultures treated with GW2580. The expression level was represented as the ratio of phosphorylated protein over the total m-CSF receptor. Statistical significance is indicated as ** *p* < 0.005, *n* = 3 cultures, *t*-test.

We screened 45 kinase inhibitors using the drug application and recording protocol shown in Figure 2. Inhibitors were screened with four cultures per inhibitor, and results were compared to four vehicle-treated cultures generated from the same animal. Seizure durations per 1 h of recording, average event rates, and electrographic loads were combined for all recordings and compared using a Kolmogorov–Smirnov test. The results for Flt-3 inhibitor [43] and cFMS receptor tyrosine kinase inhibitor (GW 2580) [44] are shown in Figure 3 as examples. These two inhibitors were also screened in our previous work [42] with similar results: cFMS inhibitor significantly reduced epileptiform activity in organotypic hippocampal cultures, while Flt-3 inhibitor had no significant effect. These results serve to validate the microwire screening platform. Western blot was conducted to confirm the suppression of kinase activity by corresponding inhibitors. Figure 3E shows a significantly decreased expression level of phospho-M-CSFR (cFMS) between control and cultures treated with cFMS inhibitor (GW2580). There was no detectable expression of phospho-flt3 in hippocampal slice cultures, which could potentially explain the lack of efficacy of Flt-3 inhibition. However, as discussed below, the lack of efficacy of a single inhibitor does not rule out Flt-3 as a potential antiepileptic drug target.

**Figure 4:**
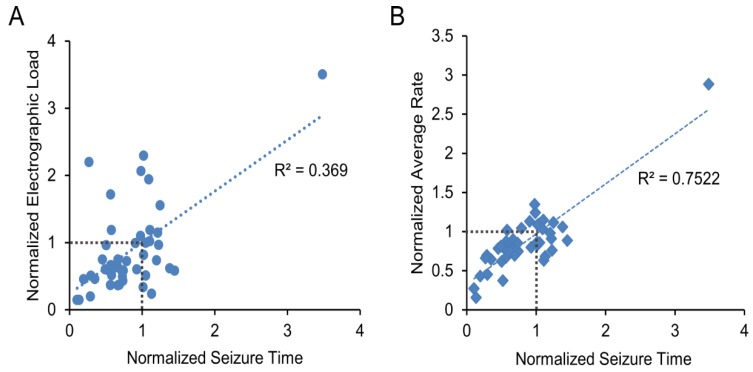
Results of the 45 inhibitor screens. (**A**) Data are plotted as normalized electrographic load versus normalized cumulative seizure time; (**B**) Data are plotted as normalized average rate versus normalized cumulative seizure time. Linear fit and Pearson correlation coefficient *R*^2^ are shown.

The results for all 45 inhibitors are shown in Table 1 and graphically represented in Figure 4. The Pearson correlation coefficient was *R*^2^ = 0.369 with *p* < 0.001 for cumulative seizure time versus electrographic load, and *R*^2^ = 0.752 with *p* < 0.001 for cumulative seizure time versus average event rate. These results indicate that while the three metrics used to evaluate the results of the screen are not completely independent of one another, cumulative seizure time and electrographic load are only weakly correlated. Thus, both should be evaluated to determine inhibitor effects.

**Figure 5:**
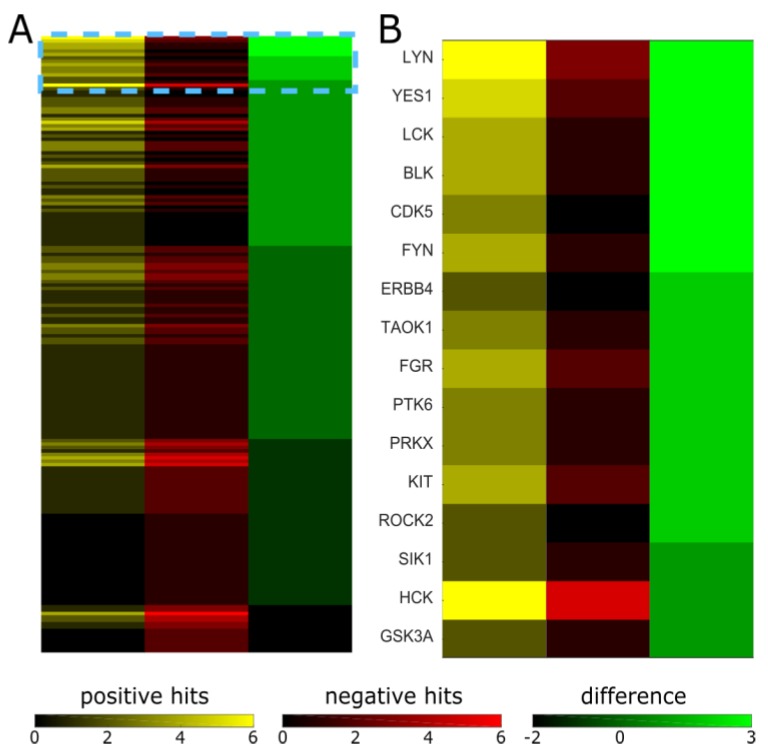
Positive and negative hits against kinases inhibited by selected inhibitors. (**A**) Map of all 182 kinases that were inhibited by one or more of the selected inhibitors: positive hits refers to the number of inhibitors that inhibited a given kinase and suppressed epileptiform activity; negative hits refers to the number of inhibitors that inhibited a given kinase but did not suppress epileptiform activity; difference refers to the difference between positive and negative hit counts. (**B**) Higher magnification view of the region indicated by a dashed box in (**A**), kinase names are shown.

Kinase inhibitors are known to not always be specific to their intended target [21,22,23]. The suppression of epileptiform activity may occur through the inhibition of the intended target or through the inhibition of off-target kinases. Inhibition profiles of many of the kinases used in our screen have been published [21] or are available through manufacturers. We used this information to determine which kinases are inhibited by the compounds that suppressed epileptiform activity, but not inhibited by the compounds that had no effect. We selected nine “positive hits” (significant inhibition of epileptiform activity) and nine “negative hits” (no significant inhibition of epileptiform activity) with known inhibition profiles, indicated in Table 1 by green and orange highlighting, respectively. We then compiled a list of 182 kinases that were inhibited at a level of 50% or higher by concentrations of inhibitors that were close to the concentrations we used in the screen. We then counted the positive and negative hits against a given kinase, and sorted results by the difference between positive and negative hits (counts) (Figure 5; complete results in Table S1).

## 3. Discussion

Six kinases (LYN, YES1, LCK, BLK, CDK5, and FYN) had three more inhibitors that targeted them and suppressed epileptiform activity than inhibitors that targeted them and did not affect epileptiform activity. Five out of six of these kinases (LYN, YES1, LCK, BLK, and FYN) are members of the Src family of non-receptor tyrosine kinases. LYN, YES1, and FYN are expressed in the human brain [45], while LYN, BLK, and FYN are expressed in the mouse hippocampus [33]. Src family kinases play an important role in learning and memory, and are involved in epilepsy [46,47]. CDK5, or cyclin dependent kinase 5, is also highly expressed in the human brain and mouse hippocampus, and plays an important role in the regulation of cytoskeletal organization, endocytosis, and exocytosis—processes important in epileptogenesis [33,45]. Thus, our approach of using the inhibition profiles of the screened inhibitors resulted in the identification of kinases that may be suitable targets for the suppression of epileptic seizures.

On the other hand, the screen also identified several kinases that were highly targeted by both effective and ineffective inhibitors of epileptiform activity. One example is NTRK2 (four positive hits, three negative hits). NTRK2 is neurotrophic receptor tyrosine kinase 2, also known as TrkB—a receptor of the brain-derived neurotrophic factor (BDNF). BDNF-TrkB signaling plays an important role in a variety of neuronal functions, and is involved in epilepsy [14,15,16,17]. Our results show that the inhibition of NTRK2 does not always reduce epileptiform activity, and that results are inhibitor-specific. Another example is CSF1R, colony stimulating factor 1 receptor, which had five positive and four negative hits on our screen. This tyrosine receptor kinase is also known as cFMS and is highly expressed in the hippocampus (Figure 1A). CSF1R is the target of GW2580, a cFMS inhibitor that significantly reduced seizure load in organotypic hippocampal cultures in our previous work [42] and in this screen (Table 1). Interestingly, cFMS inhibitor also significantly inhibits the activity of NTRK1 and NTRK2 [21]. However, several ‘negative hits’, or inhibitors that did not significantly reduce epileptiform activity, also inhibit activity of CSF1R, NTRK1, and NTRK2 (IRAK-1/4 inhibitor, VEGFR tyrosine kinase inhibitor IV, and GTP-14564). It is therefore unclear whether the inhibition of CSF1R and NTRK2 is necessary or sufficient to suppress seizures based on the results of this screen.

The approach of using published inhibition profiles to identify effective kinase targets has several limitations. Published inhibition profiles were obtained in cell-free assays whereas our screen was conducted in a tissue-based model. We selected those inhibitors that were used at somewhat higher concentrations in our screen compared to the kinase activity assays. Higher concentrations would account for a potentially reduced availability of the inhibitor within cells in our organotypic model compared to cell-free assays. The published profiles do not cover all kinases; thus, there is a possibility that seizure suppression may be occurring through the effects on unidentified components of the kinome.

## 4. Materials and Methods

### 4.1. Microwire-Integrated Culture Plate

PFA-coated tungsten wire (bare diameter = 50.8 µm, coated diameter = 101.6 µm, A-M systems Inc.) was sterilized by 70% ethanol, affixed to the substrate of a standard tissue culture 6-well plate by silicone adhesive (4300 RTV, Bluestar Silicones, Oslo, Norway), and cured at 65 °C overnight. In each culture well, a recording electrode was fixed to the substrate with the microwire tip placed in the center and a reference electrode (same type of microwire with 1.5 cm insulation layer removed at the tip) was placed on the side (Figure 2B). The microwire-integrated culture plates were then coated with poly-d-lysine (PDL, Sigma) and incubated in a humidified atmosphere at 37 °C overnight. Plates were then washed in sterile distilled water, filled with NeurobasalA/B27 medium (Thermo Fisher Scientific, Waltham, MA, USA) and incubated for at least 3 h before the placement of hippocampal slices.

### 4.2. Organotypic Hippocampal Slice Cultures

The hippocampi of postnatal day 7–8 Sprague–Dawley rat pups (Charles River Laboratories, Wilmington, MA, USA) were removed and cut into 350 μm slices on a McIlwain tissue chopper (Mickle Laboratory Eng. Co., Surrey, UK) and placed onto the microwire recording electrodes (with the electrode tip underneath the CA1/CA3 region) of the microwire-integrated culture plate, one slice per well. Slice cultures were maintained in serum-free NeurobasalA/B27 medium containing 0.5 mM glutaMAX (Thermo Fisher Scientific) and 30 mg/L gentamicin (Thermo Fisher Scientific) and incubated at 37 °C in 5% CO_2_ on a rocking platform [48]. The medium was changed twice a week. All animal use protocols were approved by the Institution Animal Care and Use Committee (IACUC) at Lehigh University (Lehigh ID 167, originally approved on 5/5/2015 and re-evaluated on a yearly basis) and were conducted in accordance with the United States Public Health Service Policy on Humane Care and Use of Laboratory Animals.

### 4.3. Electrophysiology and Data Analysis

Organotypic hippocampal cultures were maintained in microwire-integrated culture plates for two weeks. Plates were transferred to a humidified mini incubator (maintained at 37 °C and 5% CO_2_) for one-hour electrical recordings every other day (Figure 2C). Microwire electrodes were connected to a multiple-channel amplifier (RZ2, Tucker Davis Technologies) with high-impedance head stage (PZ2-64, Tucker Davis Technologies; band-pass 1 Hz-3 kHz, gain ×1000). The sampling rate was 6 kHz per channel. Signals were processed and analyzed with OpenEx (Tucker Davis Technologies) and MATLAB (MathWorks), respectively.

We extended an automated algorithm for the quantification of durations of electrographically recorded seizures [49] to also quantify the rate of paroxysmal events. Two sets of analysis were performed: (1) bin duration was set to 0.5 s to quantify the duration of seizures in the recorded time period [49], (2) bin duration was set to 0.1 s to identify paroxysmal event rates within seizures up to a maximum of 10 Hz (tonic phase of electrographic seizures) (Figure 2D,E) [50,51,52,53]. Ictal events (electrographic seizures) were defined as groups of paroxysmal events of much larger amplitude than background multiple unit activity and lasting longer than 10 s, including discrete shorter paroxysmal events that occurred with an event frequency of at least 2 Hz for at least 10 s. We used three parameters to determine the effects of inhibitors: seizure duration (time seizing during the recorded time period), average paroxysmal event rate (called event rate for conciseness), and electrographic load. Average event rate referred to the average over-threshold bin number (0.1 s binning in 10 s time window) in 45-min recordings (first 15 min of the 1-h recording was discarded). Electrographic load was the integration of the square of voltage with time.

### 4.4. Determination of Maximum Non-Toxic Inhibitor Concentration

We used the results of a lactate dehydrogenase (LDH) assay of culture supernatant and an analysis of the morphology of organotypic hippocampal cultures to determine whether an inhibitor was toxic at the applied concentration. Analyses were performed as previously described [42,48]. Briefly, all inhibitors were dissolved in DMSO and applied to *n* = 3 organotypic hippocampal slice cultures per concentration at 3 days in vitro (DIV), and morphology analysis and LDH assay were performed at 7 DIV. If LDH concentration or morphology scores were significantly elevated relative to vehicle (0.1% DMSO)-treated cultures, inhibitor concentration was considered toxic. The experiment was then repeated with a lower concentration. Maximum non-toxic concentrations of each inhibitor were used in the screen (Table 1).

### 4.5. Drug Application

Cultures from the same animal were organized into 3 experimental groups to test 2 drugs with a vehicle-treated control (*n* = 4 cultures per condition). All inhibitors were dissolved in DMSO at maximum non-toxic concentration and applied to cultures starting at 3 DIV. Control cultures were treated with 0.1% DMSO as vehicle. Inhibitors and vehicle were re-applied with each culture medium change. Electrophysiological data were then analyzed to evaluate drug efficacy. Sources of the drugs are provided in Supplementary Table S2.

### 4.6. Western Blots and Analysis

cFMS inhibitor GW2580 and Flt-3 inhibitor were applied to the culture medium after 3 days in vitro. After 24 h, cultures were collected from the 6-well plates and lysed in lysis buffer: RIPA buffer, phosphatase and protease inhibitors (Thermo Scientific, Waltham, MA, USA). Protein concentrations were assessed by micro BCA protein assay kit from Thermo Scientific. Proteins were separated in 12% Tris-Glycine Mini Gels (Life Technologies) and then transferred onto a PVDF membrane. Running and transfer buffers were purchased from Boston BioProducts. Firstly, for GW2580-treated cultures and the vehicle-treated control group the primary antibody Phospho-M-CSF receptor (Tyr723) Rabbit mAb was used for staining, and after stripping, primary M-CSF receptor antibody was applied to the membrane. For Flt-3 inhibitor-treated cultures and vehicle-treated cultures, initially primary antibody Phospho-FLT3 (Tyr589/591) Rabbit mAb was applied, and after stripping, FLT3(8F2) Rabbit mAb was applied (all primary antibodies from Cell Signaling). All primary antibodies were used at 1:1000 dilution. For all conditions Peroxidase-conjugated AffiniPure Goat Anti-Rabbit IgG (H+L) was used as the secondary antibody (Jackson ImmunoResearch). Bands were visualized on CL-XPosure X-ray films (Thermo Scientific) using SuperSignal West Femto Maximum Sensitivity Substrate. Quantification was performed using ImageJ software.

## 5. Conclusions

We developed an in vitro platform for determining the effects of compounds on chronic, spontaneous seizure-like activity. This microwire-based platform does not require sophisticated fabrication facilities and is transferrable to other laboratories. We used it to screen 45 kinase inhibitors and found several compounds with previously unknown anti-seizure effects. The examination of their kinase inhibition profiles led to the identification of kinases that may be promising targets for the development of antiepileptic drugs.

## Figures and Tables

**Table 1:** Screen summary. *p* values are from Kolmogorov–Smirnov comparison of inhibitor-treated cultures versus vehicle-treated cultures (*n* = 4 cultures, each inhibitor- and vehicle-treated control group). Green and orange highlights ‘positive hits’ and ‘negative hits’, respectively, that were selected for kinase inhibition profiling.

Drug Name	Conc. (μM)	CAS	Cumulative Seizure Duration		Average Event Rate		Electrographic Load	
			Norm.	*p*	Norm.	*p*	Norm.	*p*
Rho Kinase Inhibitor III, Rockout	10	7272-84-6	0.569	0.3	0.873	0.576	1.713	0.49
Aurora Kinase Inhibitor II	0.5	331770-21-9	0.577	0.023	0.748	0.023	1.185	0.497
GSK3, GSK3β inhibitor, Bisindolylmaleimide I	0.5	133052-90-1	0.572	0.135	0.803	0.135	0.663	0.275
Flt-3 Inhibitor	2	301305-73-7	0.656	0.387	0.856	0.051	0.664	0.622
cFMS Receptor Tyrosine Kinase Inhibitor	2	870483-87-7	0.297	0.021	0.45	0.001	0.505	0.051
EGFR/ErbB-2 Inhibitor	2	179248-61-4	0.35	0.088	0.645	0.169	0.458	0.49
GTP-14564, cFMS, c-kit, Flt3 inhibitor	5	34823-86-4	1.049	0.49	1.101	0.72	0.996	0.169
Aurora Kinase Inhibitor II	1	331770-21-9	0.742	0.6	0.747	0.89	0.58	0.72
PDGF Receptor Tyrosine Kinase Inhibitor III	2	205254-94-0	0.505	0.003	0.616	0.001	0.959	0.917
VEGF Receptor Tyrosine Kinase Inhibitor II	5	269390-69-4	0.668	0.207	0.787	0.029	0.362	0.021
VEGFR Tyrosine Kinase Inhibitor IV (Tivozanib, KRN951)	0.3	475108-18-0	1.23	0.367	0.759	0.148	0.964	0.001
PKC, PKCβ, Bisindolylmaleimide IV	10	119139-23-0	0.541	0.109	0.789	0.109	0.587	0.216
EGFR Inhibitor, BPIQ-I	10	174709-30-9	0.667	0.216	0.898	0.216	0.748	0.387
Src Kinase Inhibitor I (Src-I1)	5	179248-59-0	0.453	0.008	0.782	0.001	0.748	0.387
PKC, PKCβ, Chelerythrine Chloride	5	3895-92-9	0.927	0.861	0.798	0.387	0.599	0.109
SL0101, p90 RSK inhibitor	5	77307-50-7	1.05	0.987	0.858	0.051	0.507	0.003
GSK-3b, GSK-3β Inhibitor XI	10	626604-39-5	1.104	0.987	1.148	0.861	1.018	0.987
Ki20227, cFMS Inhibitor	5	623142-96-1	1.45	0.166	0.884	0.144	0.581	0.131
GW2580, cFMS Receptor Tyrosine Kinase Inhibitor	5	870483-87-7	0.105	0.216	0.273	0.001	0.146	0.001
SB203580, p38 MAPK Inhibitor	2.65	152121-47-6	1.248	0.861	1.116	0.622	1.554	0.109
JAK Inhibitor VI	2	856436-16-3	1.11	0.622	0.631	0.003	1.184	0.861
JAK Inhibitor I (Pyridone 6)	2	457081-03-7	1.217	0.72	0.91	0.3	1.147	0.3
CHIR99021, GSK3β Inhibitor	1	252917-06-9	0.493	0.088	0.813	0.6	0.601	0.088
EGFR Inhibitor	0.1	879127-07-8	0.788	0.72	1.04	0.003	0.724	0.088
Rho Kinase Inhibitor IV	10	913844-45-8	0.29	0.088	0.695	0.008	0.198	3.4E-4
JAK Inhibitor I	1	457081-03-7	0.716	0.3	0.728	0.088	0.589	0.088
Akt Inhibitor VIII, Isozyme-Selective, Akti-1/2	0.5	612847-09-3	0.731	0.72	0.744	0.088	0.491	0.088
Gö 6976, PKC, PKCβ	0.1	136194-77-9	0.522	0.088	0.373	1.8E-6	0.594	0.003
Akt Inhibitor V, Triciribine	0.5	35943-35-2	0.563	0.169	0.667	0.019	0.368	0.042
FR180204, MAPK (ERK1/2)	10	865362-74-9	1.2	0.711	0.979	0.994	0.736	0.162
PKCbII/EGFR Inhibitor (CGP 53353)	10	145915-60-2	0.271	0.003	0.66	0.008	2.197	2.7E-4
IRAK-1/4 Inhibitor	10	509093-47-4	0.987	0.861	1.243	0.387	2.062	0.021
PI3Kγ Inhibitor (AS 605240)	10	648450-29-7	0.979	0.995	1.348	0.169	1.099	0.169
JNK VIII inhibitor	10	894804-07-0	3.485	0.008	2.883	3.9E-7	3.502	0.001
Y-27632, ROCK Inhibitor	10	146986-50-7	1.02	1	0.832	0.49	0.813	0.49
PD 0325901, MEK Inhibitor	10	391210-10-9	0.135	2.8E-5	0.156	8.0E-8	0.144	3.4E-4
CP-690550, Tofacitinib, JAK3 Inhibitor	10	540737-29-9	0.626	0.088	0.746	0.042	0.628	0.72
BIRB 796, Doramapimod, p38 MAPK inhibitor	10	285983-48-4	0.907	0.042	1.13	0.169	0.992	0.019
BI-D1870, RSK1/2/3/4 Inhibitor	10	501437-28-1	0.194	3.4E-4	0.432	1.0E-4	0.454	0.008
AZD0530, Saracatinib, Src Inhibitor	2	379231-04-6	0.689	0.169	0.694	0.169	0.37	0.008
JNK Inhibitor IX	0.2	312917-14-9	1.131	0.188	0.682	0.023	0.237	3.7E-5
PI-103, PI3K	0.2	371935-74-9	1.094	0.088	1.034	0.49	1.939	0.088
CyclotraxinB, TRK Inhibitor	10	1203586-72-4	0.735	0.184	0.853	0.22	0.428	0.24
GNF5837, TRK Inhibitor	2	1033769-28-6	0.582	0.003	1.016	0.169	0.512	0.72
PKR Inhibitor	0.05	608512-97-6	1.016	0.917	0.864	0.72	0.334	0.3

## Supplementary Materials

Supplementary materials can be found at https://www.mdpi.com/1422-0067/20/10/2502/s1. **Supplementary Table 1.** Positive and negative hits against all analyzed kinases inhibited by screened inhibitors. **Supplementary Table 2**. Sources (vendors) of all inhibitors.

## Abbreviations

MEA	Multiple Electrode Array
RTK	Receptor Tyrosine Kinase
DIV	Days in Vitro
LDH	Lactate Dehydrogenase

## References

[1] Pitkänen A., Lukasiuk K. (2011). Mechanisms of epileptogenesis and potential treatment targets. Lancet Neurol..

[2] Goldberg E.M., Coulter D.A. (2013). Mechanisms of epileptogenesis: A convergence on neural circuit dysfunction. Nat. Rev. Neurosci..

[3] Grabenstatter H.L., Russek S.J., Brooks-Kayal A.R. (2012). Molecular pathways controlling inhibitory receptor expression. Epilepsia.

[4] Pitkänen A., Lukasiuk K. (2009). Molecular and cellular basis of epileptogenesis in symptomatic epilepsy. Epilepsy Behav. EB.

[5] Amato S., Liu X., Zheng B., Cantley L., Rakic P., Man H.-Y. (2011). AMP-activated protein kinase regulates neuronal polarization by interfering with PI 3-kinase localization. Science.

[6] Yoshii A., Constantine-Paton M. (2010). Postsynaptic BDNF-TrkB signaling in synapse maturation, plasticity, and disease. Dev. Neurobiol..

[7] Chan C.B., Liu X., Pradoldej S., Hao C., An J., Yepes M., Luo H.R., Ye K. (2011). Phosphoinositide 3-kinase enhancer regulates neuronal dendritogenesis and survival in neocortex. J. Neurosci. Off. J. Soc. Neurosci..

[8] Cuesto G., Enriquez-Barreto L., Caramés C., Cantarero M., Gasull X., Sandi C., Ferrús A., Acebes Á., Morales M. (2011). Phosphoinositide-3-kinase activation controls synaptogenesis and spinogenesis in hippocampal neurons. J. Neurosci. Off. J. Soc. Neurosci..

[9] Oliva A.A., Atkins C.M., Copenagle L., Banker G.A. (2006). Activated c-Jun N-terminal kinase is required for axon formation. J. Neurosci. Off. J. Soc. Neurosci..

[10] Berdichevsky Y., Dryer A.M., Saponjian Y., Mahoney M.M., Pimentel C.A., Lucini C.A., Usenovic M., Staley K.J. (2013). PI3K-Akt signaling activates mTOR-mediated epileptogenesis in organotypic hippocampal culture model of post-traumatic epilepsy. J. Neurosci. Off. J. Soc. Neurosci..

[11] Zeng L.-H., Rensing N.R., Wong M. (2009). The mammalian target of rapamycin signaling pathway mediates epileptogenesis in a model of temporal lobe epilepsy. J. Neurosci. Off. J. Soc. Neurosci..

[12] Buckmaster P.S., Ingram E.A., Wen X. (2009). Inhibition of the mammalian target of rapamycin signaling pathway suppresses dentate granule cell axon sprouting in a rodent model of temporal lobe epilepsy. J. Neurosci. Off. J. Soc. Neurosci..

[13] Grabenstatter H.L., Del Angel Y.C., Carlsen J., Wempe M.F., White A.M., Cogswell M., Russek S.J., Brooks-Kayal A.R. (2014). The effect of STAT3 inhibition on status epilepticus and subsequent spontaneous seizures in the pilocarpine model of acquired epilepsy. Neurobiol. Dis..

[14] Scharfman H.E. (2005). Brain-derived neurotrophic factor and epilepsy—A missing link?. Epilepsy Curr..

[15] Liu G., Gu B., He X.-P., Joshi R.B., Wackerle H.D., Rodriguiz R.M., Wetsel W.C., McNamara J.O. (2013). Transient inhibition of TrkB kinase after status epilepticus prevents development of temporal lobe epilepsy. Neuron.

[16] Aungst S., England P.M., Thompson S.M. (2013). Critical role of trkB receptors in reactive axonal sprouting and hyperexcitability after axonal injury. J. Neurophysiol..

[17] Dinocourt C., Gallagher S.E., Thompson S.M. (2006). Injury-induced axonal sprouting in the hippocampus is initiated by activation of trkB receptors. Eur. J. Neurosci..

[18] Manning G., Whyte D.B., Martinez R., Hunter T., Sudarsanam S. (2002). The protein kinase complement of the human genome. Science.

[19] Martin K.J., Arthur J.S.C. (2012). Selective kinase inhibitors as tools for neuroscience research. Neuropharmacology.

[20] Bhullar K.S., Lagarón N.O., McGowan E.M., Parmar I., Jha A., Hubbard B.P., Rupasinghe H.P.V. (2018). Kinase-targeted cancer therapies: Progress, challenges and future directions. Mol. Cancer.

[21] Gao Y., Davies S.P., Augustin M., Woodward A., Patel U.A., Kovelman R., Harvey K.J. (2013). A broad activity screen in support of a chemogenomic map for kinase signalling research and drug discovery. Biochem. J..

[22] Bain J., Plater L., Elliott M., Shpiro N., Hastie C.J., McLauchlan H., Klevernic I., Arthur J.S.C., Alessi D.R., Cohen P. (2007). The selectivity of protein kinase inhibitors: A further update. Biochem. J..

[23] Anastassiadis T., Deacon S.W., Devarajan K., Ma H., Peterson J.R. (2011). Comprehensive assay of kinase catalytic activity reveals features of kinase inhibitor selectivity. Nat. Biotechnol..

[24] Liu B., Chen H., Johns T.G., Neufeld A.H. (2006). Epidermal growth factor receptor activation: An upstream signal for transition of quiescent astrocytes into reactive astrocytes after neural injury. J. Neurosci. Off. J. Soc. Neurosci..

[25] Leadbeater W.E., Gonzalez A.-M., Logaras N., Berry M., Turnbull J.E., Logan A. (2006). Intracellular trafficking in neurones and glia of fibroblast growth factor-2, fibroblast growth factor receptor 1 and heparan sulphate proteoglycans in the injured adult rat cerebral cortex. J. Neurochem..

[26] Nagayama T., Nagayama M., Kohara S., Kamiguchi H., Shibuya M., Katoh Y., Itoh J., Shinohara Y. (2004). Post-ischemic delayed expression of hepatocyte growth factor and c-Met in mouse brain following focal cerebral ischemia. Brain Res..

[27] Schäbitz W.-R., Krüger C., Pitzer C., Weber D., Laage R., Gassler N., Aronowski J., Mier W., Kirsch F., Dittgen T. (2008). A neuroprotective function for the hematopoietic protein granulocyte-macrophage colony stimulating factor (GM-CSF). J. Cereb. Blood Flow Metab. Off. J. Int. Soc. Cereb. Blood Flow Metab..

[28] Tokita Y., Keino H., Matsui F., Aono S., Ishiguro H., Higashiyama S., Oohira A. (2001). Regulation of neuregulin expression in the injured rat brain and cultured astrocytes. J. Neurosci. Off. J. Soc. Neurosci..

[29] Sköld M.K., von Gertten C., Sandberg-Nordqvist A.-C., Mathiesen T., Holmin S. (2005). VEGF and VEGF receptor expression after experimental brain contusion in rat. J. Neurotrauma.

[30] Helmy A., Carpenter K.L.H., Menon D.K., Pickard J.D., Hutchinson P.J.A. (2011). The cytokine response to human traumatic brain injury: Temporal profiles and evidence for cerebral parenchymal production. J. Cereb. Blood Flow Metab. Off. J. Int. Soc. Cereb. Blood Flow Metab..

[31] Castañeda-Cabral J.L., Beas-Zárate C., Rocha-Arrieta L.L., Orozco-Suárez S.A., Alonso-Vanegas M., Guevara-Guzmán R., Ureña-Guerrero M.E. (2018). Increased protein expression of VEGF-A, VEGF-B, VEGF-C and their receptors in the temporal neocortex of pharmacoresistant temporal lobe epilepsy patients. J. Neuroimmunol..

[32] Song Y., Pimentel C., Walters K., Boller L., Ghiasvand S., Liu J., Staley K.J., Berdichevsky Y. (2016). Neuroprotective levels of IGF-1 exacerbate epileptogenesis after brain injury. Sci. Rep..

[33] Lein E.S., Hawrylycz M.J., Ao N., Ayres M., Bensinger A., Bernard A., Boe A.F., Boguski M.S., Brockway K.S., Byrnes E.J. (2007). Genome-wide atlas of gene expression in the adult mouse brain. Nature.

[34] Caron E., Ghosh S., Matsuoka Y., Ashton-Beaucage D., Therrien M., Lemieux S., Perreault C., Roux P.P., Kitano H. (2010). A comprehensive map of the mTOR signaling network. Mol. Syst. Biol..

[35] van der Geer P., Hunter T., Lindberg R.A. (1994). Receptor protein-tyrosine kinases and their signal transduction pathways. Annu. Rev. Cell Biol..

[36] Lemmon M.A., Schlessinger J. (2010). Cell signaling by receptor tyrosine kinases. Cell.

[37] Lim K.-C., Crino P.B. (2013). Focal malformations of cortical development: New vistas for molecular pathogenesis. Neuroscience.

[38] Jastrzebski K., Hannan K.M., Tchoubrieva E.B., Hannan R.D., Pearson R.B. (2007). Coordinate regulation of ribosome biogenesis and function by the ribosomal protein S6 kinase, a key mediator of mTOR function. Growth Factors Chur Switz..

[39] Cho C.H. (2011). Frontier of epilepsy research-mTOR signaling pathway. Exp. Mol. Med..

[40] Fernandez A.M., Torres-Alemán I. (2012). The many faces of insulin-like peptide signalling in the brain. Nat. Rev. Neurosci..

[41] Yamaguchi H., Chang S.-S., Hsu J.L., Hung M.-C. (2014). Signaling cross-talk in the resistance to HER family receptor targeted therapy. Oncogene.

[42] Liu J., Sternberg A.R., Ghiasvand S., Berdichevsky Y. (2018). Epilepsy-on-a-chip System for Antiepileptic Drug Discovery. IEEE Trans. Biomed. Eng..

[43] Patch R.J., Baumann C.A., Liu J., Gibbs A.C., Ott H., Lattanze J., Player M.R. (2006). Identification of 2-acylaminothiophene-3-carboxamides as potent inhibitors of FLT3. Bioorg. Med. Chem. Lett..

[44] Conway J.G., McDonald B., Parham J., Keith B., Rusnak D.W., Shaw E., Jansen M., Lin P., Payne A., Crosby R.M. (2005). Inhibition of colony-stimulating-factor-1 signaling in vivo with the orally bioavailable cFMS kinase inhibitor GW2580. Proc. Natl. Acad. Sci. USA..

[45] Fagerberg L., Hallström B.M., Oksvold P., Kampf C., Djureinovic D., Odeberg J., Habuka M., Tahmasebpoor S., Danielsson A., Edlund K. (2014). Analysis of the human tissue-specific expression by genome-wide integration of transcriptomics and antibody-based proteomics. Mol. Cell. Proteom. MCP.

[46] Kumar A., Jaggi A.S., Singh N. (2015). Pharmacology of Src family kinases and therapeutic implications of their modulators. Fundam. Clin. Pharmacol..

[47] Sharma S., Carlson S., Puttachary S., Sarkar S., Showman L., Putra M., Kanthasamy A.G., Thippeswamy T. (2018). Role of the Fyn-PKCδ signaling in SE-induced neuroinflammation and epileptogenesis in experimental models of temporal lobe epilepsy. Neurobiol. Dis..

[48] Berdichevsky Y., Saponjian Y., Park K.-I., Roach B., Pouliot W., Lu K., Swiercz W., Dudek F.E., Staley K.J. (2016). Staged anticonvulsant screening for chronic epilepsy. Ann. Clin. Transl. Neurol..

[49] Berdichevsky Y., Dzhala V., Mail M., Staley K.J. (2012). Interictal spikes, seizures and ictal cell death are not necessary for post-traumatic epileptogenesis in vitro. Neurobiol. Dis..

[50] Wuarin J.P., Dudek F.E. (1996). Electrographic seizures and new recurrent excitatory circuits in the dentate gyrus of hippocampal slices from kainate-treated epileptic rats. J. Neurosci. Off. J. Soc. Neurosci..

[51] Khazipov R., Khalilov I., Tyzio R., Morozova E., Ben-Ari Y., Holmes G.L. (2004). Developmental changes in GABAergic actions and seizure susceptibility in the rat hippocampus. Eur. J. Neurosci..

[52] Dzhala V.I., Talos D.M., Sdrulla D.A., Brumback A.C., Mathews G.C., Benke T.A., Delpire E., Jensen F.E., Staley K.J. (2005). NKCC1 transporter facilitates seizures in the developing brain. Nat. Med..

[53] Jirsa V.K., Stacey W.C., Quilichini P.P., Ivanov A.I., Bernard C. (2014). On the nature of seizure dynamics. Brain, J. Neurol..

